# O11.4. EDUCATION, EMPLOYMENT AND DISABILITY AMONG YOUNG PERSONS WITH EARLY PSYCHOSIS PARTICIPATING IN A COORDINATED SPECIALTY CARE PROGRAM

**DOI:** 10.1093/schbul/sby015.264

**Published:** 2018-04-01

**Authors:** Thomas Smith, Jennifer Humensky, Jennifer Scodes, Melanie Wall, Ilana Nossel, Lisa Dixon

**Affiliations:** Columbia University & New York State Psychiatric Institute

## Abstract

**Background:**

Comprehensive early treatment programs for individuals with early psychosis have demonstrated success internationally, spurring rapid expansion of the model in the United States. Between 2014–2016, U.S. federal funding to states to support Coordinated Specialty Care (CSC) for individuals with early psychosis increased to $50 million annually (Dixon, 2017). New York State (NYS) was an early adopter and has rapidly expanded CSC across the state. This study prospectively evaluated education and employment outcomes over time within NYS’s CSC program, OnTrackNY.

**Methods:**

Employment and education trajectories were assessed for individuals with early psychosis who had at least one three-month follow-up assessment, from the program’s inception in October 2013, through September 2016 (N=325). Rates of Social Security Administration (SSA) disability enrollment were assessed for individuals enrolled from October 2013 to June 2017 (n=679).

Education and employment status was estimated using longitudinal logistic models utilizing generalized estimating equations with an autoregressive covariance structure to account for within-subject correlations over time. To test how education/employment changed over time, pre-specified contrasts were tested from the longitudinal model for the mean change in sequential follow-up visits. A Kaplan-Meier estimator with discrete time to event and censoring at last observed follow-up month with no event was used to estimate the probability of any education/employment by one year after admission and to estimate the risk of disability by two years after admission.

**Results:**

Approximately 40% of individuals with early psychosis were engaged in school or work upon enrollment in a CSC program; engagement increased to 80% after 6 months of care. The estimated probability of being employed or in school at some time during the year after admission was 87.9% (95% Confidence Interval (CI)= [82.9, 92.0]). Relative to women, men had significantly lower odds of education/employment. Relative to non-Hispanic whites, individuals who were Asian, Hispanic or Black had lower odds of education/ employment. Relative to individuals who had not yet completed high school, individuals whose highest educational attainment was High School (HS) or GED had lower odds of educational/employment.

At admission, 2.5% (17/679) clients were receiving SSA disability benefits. The Kaplan-Meier estimates that 18.3% (95% CI= [13.9, 23.9]) of clients followed for two years obtained disability benefits. In bivariate cox regression analyses, individuals with lower (worse) occupational and social functioning scores have significantly greater risk of disability enrollment than individuals with higher scores (in multivariate analysis, only lower occupational functioning remains significant). Age, gender, race, ethnicity, and symptom scores were not significantly associated with disability enrollment.

**Discussion:**

This study demonstrates that individuals with early psychosis who receive CSC in non-research community settings achieve significant improvements in education and employment. Gender, race/ethnicity, and baseline education predicted education and employment outcomes, while poorer functioning was associated with risk of SSA disability benefits. CSC teams should make particular efforts to support the work and school goals of individuals who may be more likely to struggle in achieving engagement in work and school.

